# Influence of trap type on the captures of *Lymantria dispar* L. (Lepidoptera: Erebidae): trials from different European countries

**DOI:** 10.1093/jee/toae223

**Published:** 2024-11-09

**Authors:** Paraskevi Agrafioti, Evagelia Lampiri, Tanja Bohinc, Anna Roig, Alexandre Levi-Mourao, Maria C Boukouvala, Anna Skourti, Carmen López, Matilde Eizaguirre, Xavier Pons, Angelos Tsikas, Ankica Sarajlić, Jozsef Fail, Enrique Benavent Fernandez, Sergio Fita Bravo, Elena Dominguez Solera, Stanislav Trdan, Nickolas G Kavallieratos, Christos G Athanassiou

**Affiliations:** Department of Agriculture, Laboratory of Entomology and Agricultural Zoology, Crop production and Rural Environment, University of Thessaly, Nea Ionia, Greece; Department of Agriculture, Laboratory of Entomology and Agricultural Zoology, Crop production and Rural Environment, University of Thessaly, Nea Ionia, Greece; Department of Agronomy, Biotechnical Faculty, University of Ljubljana, Ljubljana, Slovenia; PROBODELT, Pest Control Company, Tarragona, Spain; Department of Crop and Forest Sciences, Agrotecnio Centre, Universitat de Lleida, Lleida, Spain; Department of Crop Science, Laboratory of Agricultural Zoology and Entomology, Agricultural University of Athens, Athens, Greece; Department of Crop Science, Laboratory of Agricultural Zoology and Entomology, Agricultural University of Athens, Athens, Greece; Department of Crop and Forest Sciences, Agrotecnio Centre, Universitat de Lleida, Lleida, Spain; Department of Crop and Forest Sciences, Agrotecnio Centre, Universitat de Lleida, Lleida, Spain; Department of Crop and Forest Sciences, Agrotecnio Centre, Universitat de Lleida, Lleida, Spain; Department of Forestry and Management of the Environment and Natural Resources, Laboratory of Forest Protection, School of Agricultural and Forestry Sciences, Democritus University of Thrace, Komotini, Greece; Faculty of Agrobiotechnical Sciences Osijek, Josip Juraj Strossmayer, University of Osijek, Osijek, Croatia; Hungarian University of Agriculture and Life Sciences, Institute for Plant Protection, Budapest, Hungary; AIMPLAS, Plastics Technology Centre, València Parc Tecnològic, Valencia, Spain; AIMPLAS, Plastics Technology Centre, València Parc Tecnològic, Valencia, Spain; AIMPLAS, Plastics Technology Centre, València Parc Tecnològic, Valencia, Spain; Department of Agronomy, Biotechnical Faculty, University of Ljubljana, Ljubljana, Slovenia; Department of Crop Science, Laboratory of Agricultural Zoology and Entomology, Agricultural University of Athens, Athens, Greece; Department of Agriculture, Laboratory of Entomology and Agricultural Zoology, Crop production and Rural Environment, University of Thessaly, Nea Ionia, Greece

**Keywords:** *Lymantria dispar*, spongy moth, pheromone traps, monitoring, trap type

## Abstract

The spongy moth, *Lymantria dispar* L. (Lepidoptera: Eribidae), is a serious pest of deciduous forests and causes widespread defoliation. Despite this, few studies have evaluated the wide-ranging surveillance of adult male *L*. *dispar* using different types of pheromone-baited traps. We evaluated the effect of trap type on captures of adult male *L. dispar* at 18 sites in Europe; two in Slovenia, two in Spain, 12 in Greece, one in Hungary, and one in Croatia. Seven different trap types, G trap and eGymer 1–6, were evaluated June–September 2022 and 2023. Generally, captures of *L*. *dispar* started in late June and lasted until mid-August. Trap type affected captures. The G trap (consisting of a dark brown plastic rectangular parallel-piped body) caught significantly more *L*. *dispar* than other trap types in many instances, particularly when the peak of the flight period occurred. Captures of *L. dispar* in pairs of different trap types showed a significant correlation in trap catch in most investigations, suggesting that most detected comparable fluctuations in *L*. *dispar* abundance. We recommend that the G trap be used for wide-ranging surveillance of *L. dispar* in Europe.

## Introduction

The spongy moth, formerly known as the gypsy moth, *Lymantria dispar* L. (Lepidoptera: Erebidae), is a species of Eurasian origin that has spread throughout large areas of the Nearctic zone (Boukouvala et al. 2022). The species has a wide range of food preferences but is mostly known as a pest of deciduous trees. *Lymantria dispar* can cause severe defoliation, declines in the health of infested trees, and mortality of repeatedly infested trees (Boukouvala et al. 2022). *Lymantria dispar* is univoltine and overwinters as egg masses. Upon hatching, larvae, move to the tree canopy, usually in spring, where they complete their life cycle (Zharkov and Tvaradze 1988, Fabel 2000). Adults are active during the warm period of the year. Females produce large numbers of eggs for several weeks (Leonard 1968, 1974, NO LABEL 1981, Grijpma 1989, Sawyer et al. 1993, Pogue and Schaefer 2007).

The female sex pheromone of *L. dispar* is (7R, 8S)-cis-2-methyl-7, 8-epoxyoctadecane, (+)-disparlure. Disparlure has been frequently used for monitoring of adult male *L. dispar* (Bierl et al. 1970, Yamada et al. 1976). Elkinton and Liebhold (1990) used an extensive set of data from pheromone-baited traps to illustrate the occurrence and distribution of *L. dispar* in North America, while Leonhardt et al. (1990, 1992) evaluated the efficacy of disparlure on captures of *L. dispar*, along with factors that affected the release of disparlure over time. Moreover, Thorpe et al. (1993) used *L*. *dispar* captures in pheromone-baited traps to predict population levels. More recently, Tobin et al. (2009) used disparlure in automated traps for real-time monitoring. Based on the above, traps baited with disparlure are useful tools for early detection and area-wide monitoring of *L. dispar*, especially in newly invaded and adjacent areas.

Despite the fact that pheromone-baited traps have been extensively used in plant protection as a means of time control measures, data obtained from these traps may vary by trap type. For example, Athanassiou et al. (2002) tested different trap designs for the capture of cotton pink bollworm, *Pectinophora gossypiella* (Saunders) (Lepidoptera: Gelechiidae), and found that funnel traps were able to capture many more male adults than traps with sticky surfaces. More recently, for the pine processionary moth, *Thaumetopoea pityocampa* (Dennis and Schiffermüller) (Lepidoptera: Thaumetopoeidae), an extensive trap comparison conducted in Greece, Italy, and Spain indicated that some trap types provided better results than others, and that trap performance differed among geographic locations (Athanassiou et al. 2017). Similar results have been observed for *L. dispar*. For example, Cardé et al. (2018) compared delta traps with bucket traps and found that delta traps generally provided more consistent results, but there were strong interactions between *L*. *dispar* captures and pheromone concentrations. Moreover, Taylor et al. (1991) reported that *L. dispar* captures in the so-called “milk-carton” pheromone-baited traps correlated well with captures in suction traps, but underlined the need to obtain standardized results, which should be based on specific trapping protocols. Elkinton and Childs (1983) found that commercial sticky traps were more effective than the “milk-carton” traps, but that trap placement influenced captures among trap types.

The majority of the above studies are based on data obtained from pheromone-baited traps evaluated in a single experimental area. Since geographic area is an important parameter in trapping performance (Athanassiou et al. 2017), we extensively compared different trap types for the capture of *L*. *dispar* at 18 sites in four European countries during two consecutive years (2022 and 2023).

## Materials and Methods

Two different protocols were implemented in different geographic areas, one called limited trap comparison and the other complete trap comparison.

### Experimental Sites for Limited Trap Comparison

In 2022, the limited trap comparison experiments were conducted at six different sites, two in Slovenia (Ginjevec 1 and Ginjevec 2), two in Spain (Montnegre-Corredor and Les Gavarres), and two in Greece (Pindos and Drama), while in 2023 experiments were conducted in these and two additional sites in Greece (Petralona 1 and Petralona 2). More information regarding the coordinates, region, experimental period, total area (ha), trees/ha, tree species, tree height (m), climate, average minimum temperature for June–August, average maximum temperature for June–August, and average rainfall for June–August for each site is provided in Table 1. The selection of experimental areas was based on the detection of *L. dispar* egg masses during the spring.

**Table 1.** Experimental sites characteristics for the limited and complete trap comparisons in 2022 and 2023

Protocol	Country	Sites	Coordinates	Region	Period	Total area (ha)	Trees/ha	Tree species	Tree height (m)	Climate	Average minimum temperature for June–August (°C)	Average maximum temperature for June–August (°C)	Average rainfall for June–August (mm)
Limited trap comparison	Slovenia	Ginjevec 1	46º37ʹ32.8″N, 16º21ʹ10.0″E	Prekmurje, NE Slovenia	2022–2023	380	100	*Quercus rubur* L. (Fagales: Fagaceae), *Carpinus betulus* L. (Fagales: Betulaceae), *Pinus sylvestris* L. (Pinales: Pinaceae)	20–30	Pannonian	15.8	27.4	124.7
	Slovenia	Ginjevec 2											
	Spain	Montnegre-Corredor	41º37ʹ27.4″N, 2º24ʹ14.6″E	Barcelona, Catalonia	2022–2023	15,000	500	*Quercus suber* L. (Fagales: Fagaceae)*, Quercus ilex* L. (Fagales: Fagaceae) *Arbutus unedo* L. (Ericales: Ericaceae)	6–8	Mediterranean	16.5	29.7	33.9
	Spain	Les Gavarres	41º840925″N, 2º9568410″E	Girona, Catalonia		28,000					14.3	28.9	33.3
	Greece	Pindos	39°48ʹ41.16″N, 20°43ʹ54.93″E	Rontovani Central Zagori, Epirus	2022–2023	159	120	*Quercus coccifera* (L.) (Fagales: Fagaceae), *Cornus mas* (L.) (Cornales: Cornaceae), *C. betulus Prunus spinosa* (L.) (Rosales: Rocaceae)	<6	Mediterranean	9.1	31.9	57.7
	Greece	Drama	39°23ʹ28″N 22°59ʹ43“E	Petrousa, Prosotsani		10	80–100	*Q. coccifera*	5–20	Mediterranean	13.7	37.3	28.4
	Greece	Petralona 1	37°43128″N 21°81144″E	Ileia, Peloponnese	2023	10.9	100	*Quercus* spp.	1–6	Mediterranean	19.1	32.9	0.2
	Greece	Petralona 2	37°43859″N 21°82632”E			7.5							
Complete trap comparison	Greece	Kouri	39°11ʹ46″N 22°44ʹ03″E	Almyros, Magnesia	2022–2023	150	80–100	*Quercus* spp.	5–20	Mediterranean	16.4	36	26.6
	Greece	Portaria	39°23ʹ28″N 22°59ʹ43″E	Pelion, Magnesia		10					16.8	33.8	12.6
	Greece	Lykodromio	41°23ʹ10.14″N 24°48ʹ7.45″E	Xanthi, Thrace	2022–2023	8.2	80–100	*Quercus frainetto* (Ten.) (Fagales: Fagaceae), *Quercus pendiculiflora* (L.) (Fagales: Fagaceae) *Carpinus orientalis* Mill. (Fagales: Betulaceae)	5–20	Mediterranean	15.5	36.6	24
	Greece	Stavroupoli	41°12ʹ10.17″N 24°42ʹ54.66″E			6.2		*Q. coccifera*			10.4	38.2	35.4
	Greece	Kryoneri Cemetery	37°46ʹ19.8″N 21°79ʹ99.1″E	Ileia, Peloponnese	2022	7.1	100	*Quercus* spp.	1–6	Mediterranean	19.1	32.9	0.2
	Greece	Kryoneri 1	37°45ʹ97.3″N 21°80ʹ14.9″E		2022–2023	6.5							
	Greece	Kryoneri 2	37°44ʹ86.1″N 21°79ʹ31.1″E			6.4							
	Greece	Saint Nickolas	37°44ʹ47.5″N 21°79ʹ09.6″E		2022	6.9							
	Hungary	Telki	47°56ʹ04.57″N 18°84ʹ05.48″E	Budai-hegység, Telki	2023	9,517	1,400	*Quercus cerris* (L.) (Fagales: Fagaceae), *Quercus petraea* (Matt.) Liebl. (Fagales: Fagaceae), *Acer campestre* (L.) (Sapindales: Sapindaceae), *Ulmus minor* Mill. (Rosales: Ulmaceae), *Fraxinus ornus* (L.) (Lamiales: Oleaceae), *Fraxinus excelsior* (L.) (Lamiales: Oleaceae)	3–11	Temperate seasonal	16.7	24.5	59
	Croatia	Koška	45°57ʹ42.95″N 18°26ʹ75.90″E	Osijek-Baranja, Eastern Croatia		826	–	*Q. robur*	33	Moderate continental	15.9	27.9	41

### Experimental Sites for Complete Trap Comparison

In 2022, the complete trap comparison experiments were conducted at eight sites in Greece. The first two sites were in the Magnesia Region (Kouri and Portaria); the second sites were in the Xanthi Region (Lykodromio and Stavroupoli); and the other four sites were in the Ileia Region (Kryoneri Cemetery, Kryoneri-Trianta Road 1, Kryoneri-Trianta Road 2, and Trianta Saint Nickolas). In 2023, two sites were added, one in Hungary (Telki) and one in Croatia (Koška), while the Kryoneri Cemetery and Trianta Saint Nickolas sites were not used. More information regarding these sites is provided in Table 1.

### Trap Types

Seven trap types were examined: G trap, eGymer 1, eGymer 2, eGymer 3, eGymer 4, eGymer 5, and eGymer 6. The G trap (SANIDAD AGRÍCOLA ECONEX, S.L.) has been described in detail by Athanassiou et al. (2017). In brief, it consists of a dark brown plastic rectangular parallel-piped body, one elastic band, and one insect reservoir (plastic bag). The upper part of the G trap is transparent and the lower part is black (Fig. 2A). eGymer 1 is the typical funnel trap in white color (PROBODELT, Amposta, Spain), the inner walls of which were coated with 45 mg of lambda-cyhalothrin (Karate Zeon 10% CS, Syngenta, Madrid, Spain), which corresponds to 0,45 ml of Karate Zeon. At the center of the trap’s disc, there is a hole (0.4 cm diameter) into which the metal cord enters, allowing the trap to be hung (Fig. 2B-E). Disparlure is placed inside the trap through the body opening. Moths enter the body through its opening, come into contact with the insecticidal agent, and die inside the container. eGymer 2 is molded from red plastic and has the following dimensions: trap length: 29 cm, trap center diameter: 14 cm, funnel diameter at the left and right ends of the trap: 11.5 cm, funnel entrance hole diameter: 3 cm (Fig. 2F and G). The trap is made up of 2 equal cylindrical parts, each containing a funnel at the bottom of the cylinder. The two parts are connected by a hinge function at the upper end of the cylinder. The eGymer 2 trap can be opened by unfolding the two parts. On the opposite side of the hinge function, there is a locking mechanism with a plastic band which is used to lock the two parts of the trap. A hook allows the eGymer 2 trap to be placed in a horizontal position, which provides 2 diagonal entrances for insects to enter the trap through the funnels. Once inside the trap, insects contact the insecticidal agent which leads to their death. eGymer 3 is made from the same materials and components as eGymer 2, with the difference being the trap system consists of 4 red cylindrical containers with a funnel at the lower end, rather than 2 (total trap length: 42 cm, trap center diameter: 14 cm, funnel diameter at the left, and right ends of the trap: 11.5 cm, funnel entrance hole diameter: 3 cm) (Fig. 2H and I). The two other parts of the trap are stacked on the two inner cylinders on the left and right sides of the funnel opening. This provides a horizontal entrance on both sides, which leads through 2 funnels on each side into the inside of the trap. eGymer 4 has the same characteristics and consists of the same components as eGymer 3 with the difference being that the four cylindrical components with funnel openings at the lower end are molded from brown plastic and that the inner walls of the trap were covered with lambda-cyhalothrin (Fig. 2J and K). eGymer 5 is identical in its composition to eGymer 4 (Fig. 2L and M), with the sole difference being that eGymer 5 does not contain the insecticidal toxicant. eGymer 6 is identical to eGymer 4 (Fig. 2N and O) but with 45 mg of deltamethrin (Delmur 2,5% EC, Sarabia, Spain), which corresponds to 1,80 ml of Delmur applied to the middle part of the trap as a toxicant. In summary, eGymer 1 and eGymer 4 were coated inside with lambda-cyhalothrin and eGymer 6 with deltamethrin, while all the other traps were free of insecticides. All trap types were baited with dispalure (purchase: May 2022) containing 1 mg of the sex pheromone component Z 7,8 epoxy-2-methyloctadecane (Novagrica, Attica, Greece).

### Placement and Inspection of Traps

In 2022, all experimental sites of the limited trap comparison evaluated the G trap, eGymer 1 and eGymer 2, while in 2023 the G trap, eGymer 3 and eGymer 4 were evaluated. Twelve traps were included at each location since there were four blocks and one trap of each type in each block (four replicates for each trap type). For the complete trap comparisons in Greece, four trap types were evaluated in 2022 (G trap, eGymer 1, eGymer 2, and eGymer 3) and 2023 (G trap, eGymer 4, eGymer 5, and eGymer 6), with one block for each site which included one trap of each type; therefore, there were 4 traps for each location. For the 2023 complete trap comparison in Hungary and Croatia, G trap, eGymer 1 and eGymer 2 were tested, with three blocks for each site, and each block included one trap of each type; therefore, there were 6 traps for each location (three replicates for each trap type). Blocks were separated by >100 m and traps within blocks were separated by ~100 m. Traps were left hanging in the experimental sites from late June to the final week of August or till early September, following the flight activity of *L. dispar*. All traps were inspected for captured *L*. *dispar* every 3–7 d. Moths tallied on each collection date were removed from traps. After the termination of this procedure, the traps were rotated clockwise within each block to minimize the influence of the individual trapping location (Athanassiou et al. 2002). Disparlure was replaced every month.

### Statistical Analysis

For the limited trap comparison, a two-way ANOVA was performed, separately for each experimental period (2022 and 2023) and each site with date and trap type, as main effects. To locate the differences among the trap types, one-way ANOVA was used for each check trap date in each site. Means were separated by the Tukey–Kramer (HSD) test at 0.05 probability (Sokal and Rohlf 1995). To examine relationships in the fluctuation of captures between trap types, independently for each site and time, correlation coefficient values between trap types were determined. To assess if these values deviated from zero, the two-tailed *t*-test was performed with a probability of 0.05 and n -2 *df*. For the complete trap comparison data, statistical analyses were not conducted due to the lack of replication, except for Telki and Koska data, for which the same statistical procedure as limited trap comparison data was followed.

## Results

### Limited Trap Comparison

#### Slovenia

**Fig. 1.**
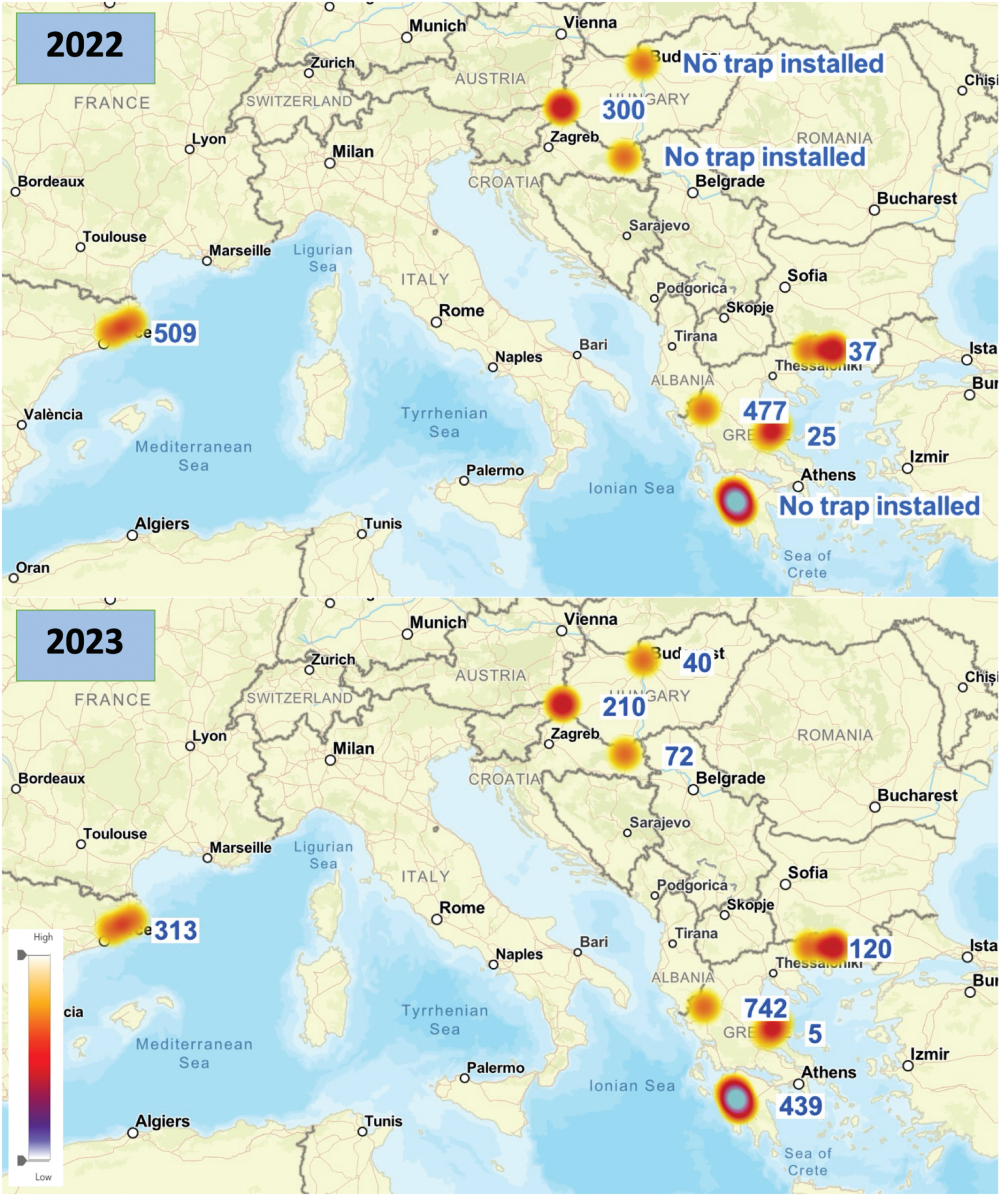
Population density of *L. dispar* at the 2022 and 2023 experimental periods in the examined areas.

In total, 300 and 552 *L. dispar* were captured in the different trap types at Ginjevec 1 and Ginjevec 2 in 2022 and 2023 (Fig. 1). In both sites and years, the flight period started at the end of June and lasted until mid-August (Fig. 3). All effects and their interactions were significant in 2022 with the exception of Trap*Site, while in 2023 only the main effects were significant (Table 2). The G trap captured significantly more *L*. *dispar* than eGymer 1 and eGymer 2 in 2022, while no differences were observed among trap types in 2023 (Fig. 3). Pairs of trap types showed positive and significant correlation coefficient values for all trap combinations in 2022, while in 2023 only G trap—eGymer 3 had a significant correlation coefficient (Table 3).

**Table 2.** ANOVA parameters for main effects and their interaction of catches of *Lymantria dispar* male adults in traps for the experimental areas in 2022 and 2023

	Slovenia						Spain						Greece					
	2022			2023			2022			2023			2022			2023		
	*df*	F	*P*	*df*	F	*P*	*df*	F	*P*	*df*	F	*P*	*df*	F	*P*	*df*	F	*P*
Date	12	20.75	**<0.01**	14	5.97	**<0.01**	10	13.98	**<0.01**	11	19.87	**<0.01**	11	31.08	**<0.01**	23	27.01	**<0.01**
Trap	2	31.18	**<0.01**	2	8.15	**<0.01**	2	26.36	**<0.01**	2	21.25	**<0.01**	2	3.17	**0.04**	2	1.73	0.17
Site	1	12.70	**<0.01**	1	4.42	**0.03**	1	0.04	0.84	1	33.48	**<0.01**	1	0.00	0.96	3	47.98	**<0.01**
Date*Trap	24	6.05	**<0.01**	26	1.25	0.19	20	2.84	**<0.01**	22	3.66	**<0.01**	22	2.66	**<0.01**	46	1.50	**0.02**
Date*Site	12	5.56	**<0.01**	13	1.47	0.12	10	1.80	0.06	11	7.30	**<0.01**	5	0.00	1.00	24	12.58	**<0.01**
Trap*Site	2	2.66	0.07	2	2.75	0.06	2	8.59	**<0.01**	2	10.62	**<0.01**	2	0.00	0.99	6	3.30	**<0.01**
Date*Trap*Site	24	2.68	**<0.01**	26	1.37	0.11	20	1.67	**0.03**	22	1.78	**0.02**	10	0.01	1.00	48	1.02	0.43

For Slovenia total *df* = 312 and 335 for 2022 and 2023, respectively, for Spain total *df* = 262 and 288 for 2022 and 2023, respectively, for Greece total *df* = 216 and 612 for 2022 and 2023, respectively, Tukey HSD test at 0.05.

**Table 3.** Correlation coefficient values (*r*) for captures between pairs of different trap types during the 2022 and 2023 monitoring period

		Slovenia		Spain		Greece	
	Pair of traps	R	*P*	R	*P*	R	*P*
2022	G trap—eGymer 1	0.44^a^	**<0.01**	0.28^a^	**<0.01**	0.72	0.12
	G trap—eGymer 2	0.54^a^	**<0.01**	0.36^a^	**<0.01**	0.72	0.54
	eGymer 1—eGymer 2	0.67^a^	**<0.01**	0.42^a^	**<0.01**	0.77	0.15
2023	G trap—eGymer 3	0.18^a^	**<0.01**	0.83^a^	**<0.01**	0.41	0.49
	G trap—eGymer 4	0.06	0.21	0.76^a^	**<0.01**	0.46	0.41
	eGymer 3—eGymer 4	0.07	0.02	0.64	0.40	0.65	0.09

^a^An asterisk declares that the value is significantly different from 0, for Slovenia *df* = 103 and 111 for 2022 and 2023, respectively, for Spain *df* = 69 and 47 for 2022 and 2023, respectively, for Greece *df* = 71 and 204 for 2022 and 2023, respectively, two-tailed *t*-test at 0.05.

**Fig. 2.**
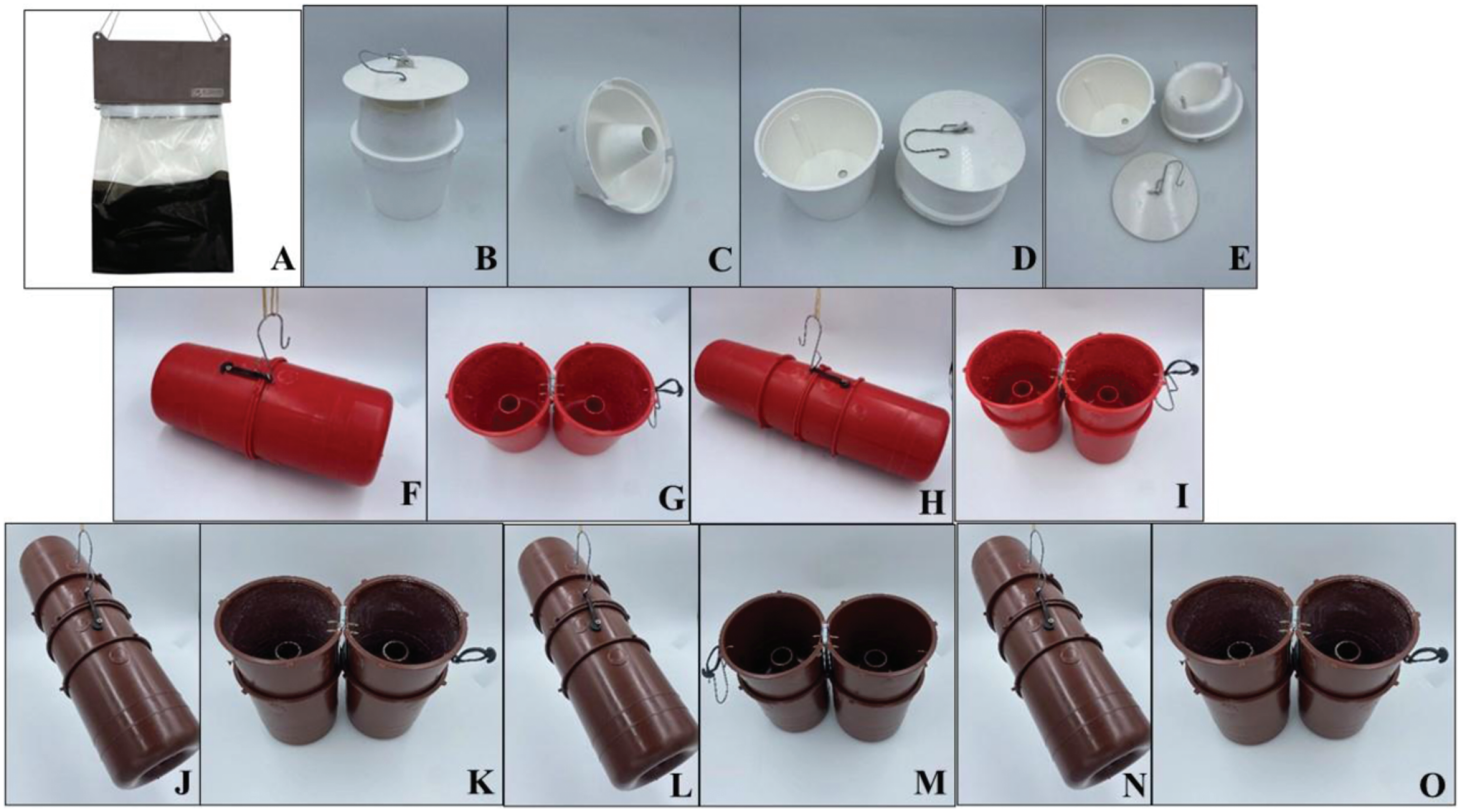
Trap devices that were tested and their parts: A) G trap fully composed, B) eGymer1 fully composed, C) funnel part of eGymer 1, D) Left: Base eGymer1/right: Funnel part and lid assembled eGymer1, E) eGymer1 in parts, F) eGymer2 fully composed, G) eGymer2 unfolded, view of the internal part with 2 funnels, H) eGymer3 fully composed, I) eGymer3 unfolded, view of the internal part showing 2 of 4 tunnels, J) eGymer4 fully composed, K) eGymer 4 unfolded, view of the internal part showing 2 of 4 tunnels funnels, L) eGymer5 fully composed, M) eGymer5 unfolded, view of the internal part showing 2 of 4 funnels, N) eGymer6 fully composed O) eGymer6 unfolded, view of the internal part showing 2 of 4 funnels.

**Fig. 3.**
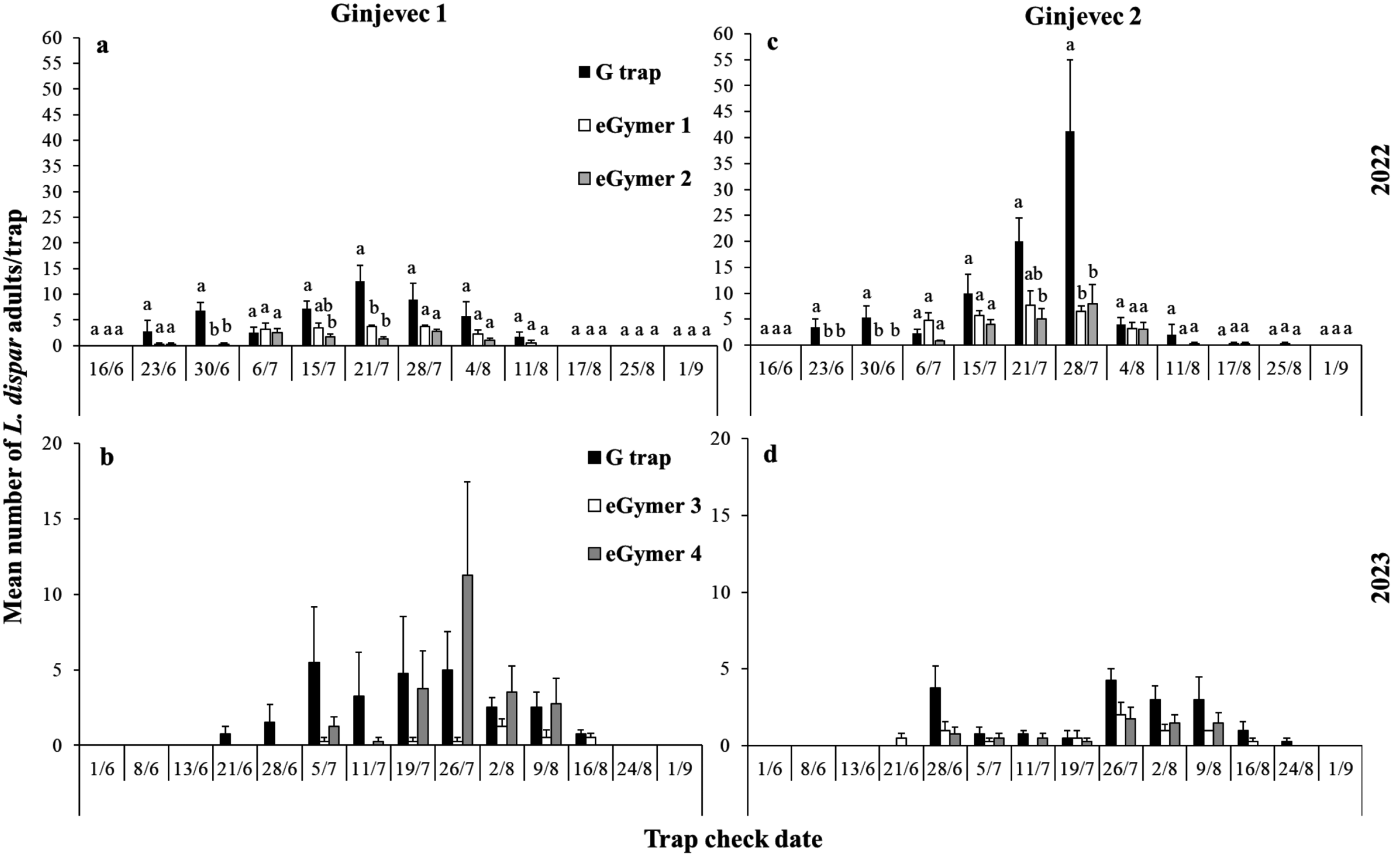
Mean number (±SE) of male adults of *Lymantria dispar* captured in each trap type and each collection date in Ginjevec 1 for 2022 A) and 2023 B) and in Ginjevec 2 for 2022 C) and 2023 D) experimental period. Within each collection date, means followed by the same letter were not significantly different (in all cases *df *= 2.11). Where no letters exist, no significant differences were noted. ANOVA parameters for Ginjevec 1 for 2022 at 30/6 were: F = 15.73, *P* < 0.01, at 15/7 were: F = 7.38, *P = *0.01, at 21/7 were: F = 10.09, *P* < 0.01, for Ginjevec 2 for 2022 at 23/6 were: 5.44, *P = *0.02, at 30/6 were: F = 5.27, *P = *0.03, at 21/7 were: F = 5.99, *P = *0.02, at 28/7 were: F = 5.74*, P = *0.02.

#### Spain

**Fig. 4.**
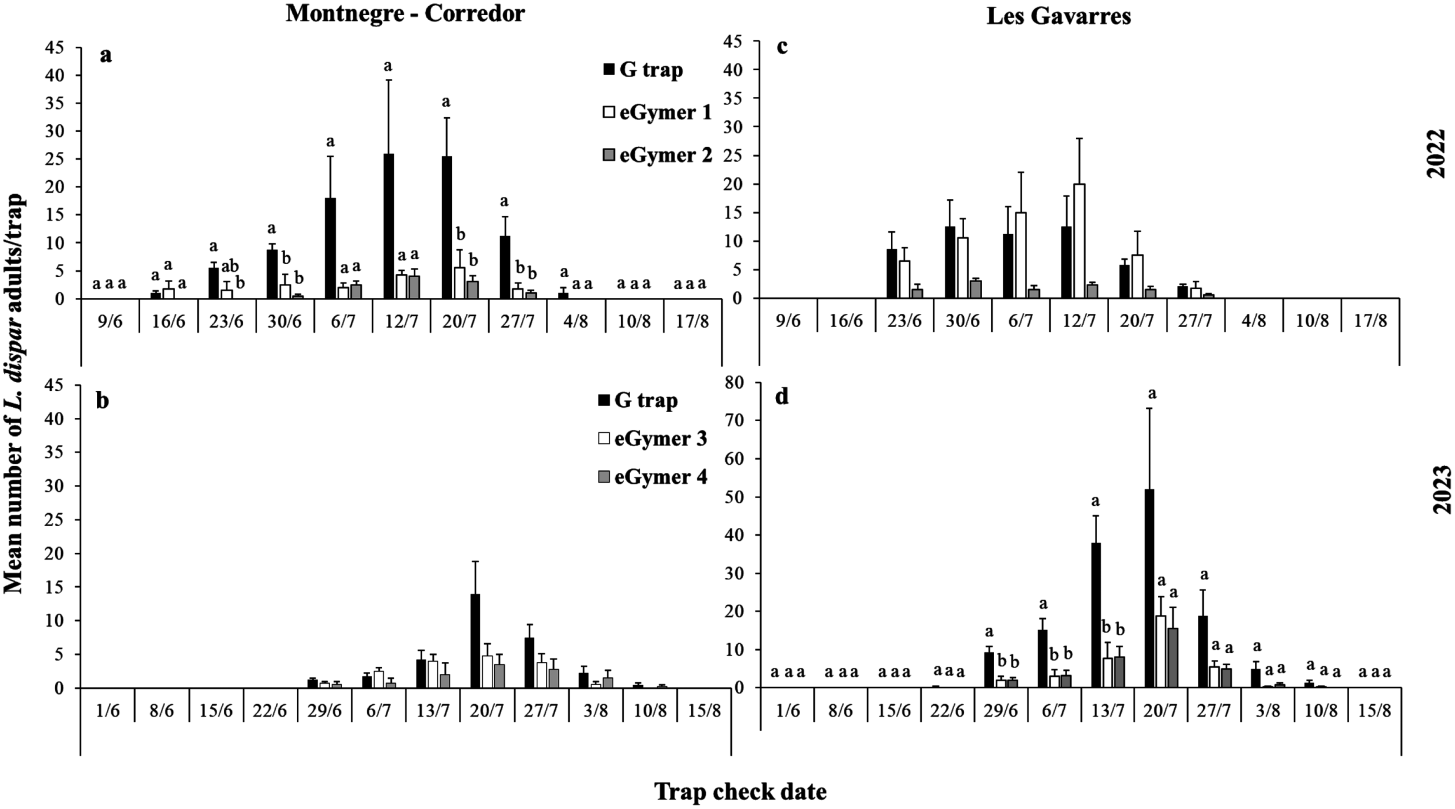
Mean number (±SE) of male adults of *Lymantria dispar* captured in each trap type and each collection date in Montnegre-Corredor for 2022 A) and 2023 B) and in Les Gavarres for 2022 C) and 2023 D) experimental period. Within each collection date, means followed by the same letter were not significantly different (in all cases *df *= 2.11). Where no letters exist, no significant differences were noted. ANOVA parameters for Montnegre-Corredor for 2022 at 23/6 were: F = 7.27, *P = *0.01, at 30/6 were: F = 11.34, *P < *0.01, at 20/7 were: F = 7.66, *P = *0.01, at 27/7 were: F = 7.10*, P = *0.01, for Les Gavarres for 2023 at 29/6 were: F = 12.42, *P < *0.01, at 6/7 were: F = 11.49, *P < *0.01, at 13/7 were: F = 12.19, *P < *0.01.

In total, 509 and 313 *L*. *dispar* were captured in Montnegre-Corredor in 2022 and 2023, while 1193 and 496 *L*. *dispar* were captured in Les Gavarres in 2022 and 2023 (Fig. 1). In 2022, the flight period started in mid-June and continued until the first week of August (Fig. 4A and C). In 2023, the flight period started in late June and continued through 10 August (Fig. 4B and D). Collection date, trap type, site, and their interactions affected the number of *L*. *dispar* captured in the traps in 2022, while in 2023 site and site*date were the only effects that were not significant (Table 2). The G trap captured significantly more *L*. *dispar* than eGymer 1 and eGymer 2 in 4 out of 8 collection dates at Montnegre-Corredor in 2022 (Fig. 4A), while in 2023 the G trap captured significantly more *L*. *dispar* than eGymer 3 and eGymer 4 in 3 out of 12 collection dates at Les Gavarres (Fig. 4D). No significant differences were observed among trap types at Les Gavarres in 2022 or at Montnegre-Corredor in 2023 (Fig. 4B and C). The correlation coefficient values were positive and significant for all pairs of traps in 2022 (Table 3) and in the 2023 period, except for eGymer 3—eGymer 4 (Table 3).

#### Greece

**Fig. 5.**
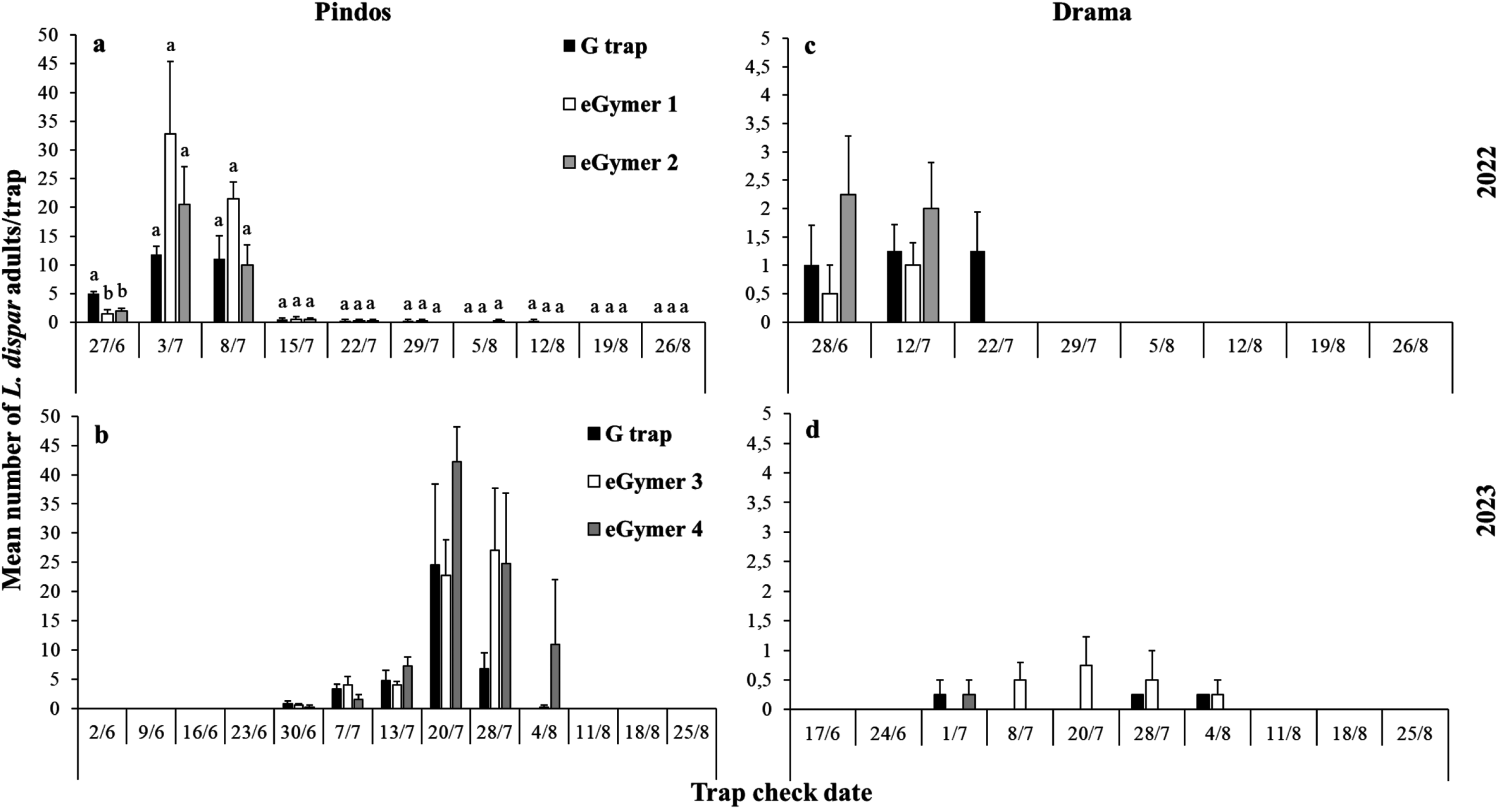
Mean number (±SE) of male adults of *Lymantria dispar* captured in each trap type and each collection date in Pindos for 2022 A) and 2023 B) and in Drama for 2022 C) and 2023 D) experimental period. Within each collection date, means followed by the same letter were not significantly different (in all cases *df *= 2.11). Where no letters exist, no significant differences were noted. ANOVA parameters for Pindos for 2022 at 27/6 were: F = 14.33, *P < *0.01.

In total, 37 and 120 and 477 and 742 *L. dispar* were captured in the different trap types at Drama and Pindos in 2022 and 2023, respectively (Fig. 1). The flight period in 2022 started at the end of June and lasted to mid-August in Pindos, while in Drama the flight period started at the end of June but lasted until late July (Fig. 5A and C). In 2023, the flight period started at the end of June and lasted until early August at both sites (Fig. 5B and D). Collection date and trap type were significant in 2022, while most of the main effects and their interactions were significant in 2023(Table 2). The G trap captured significantly more *L*. *dispar* than eGymer 1 and eGymer 2 in Pindos on 27 June 2022 (Fig. 5A). The correlation coefficient values were positive but insignificant (Table 3).

**Fig. 6.**
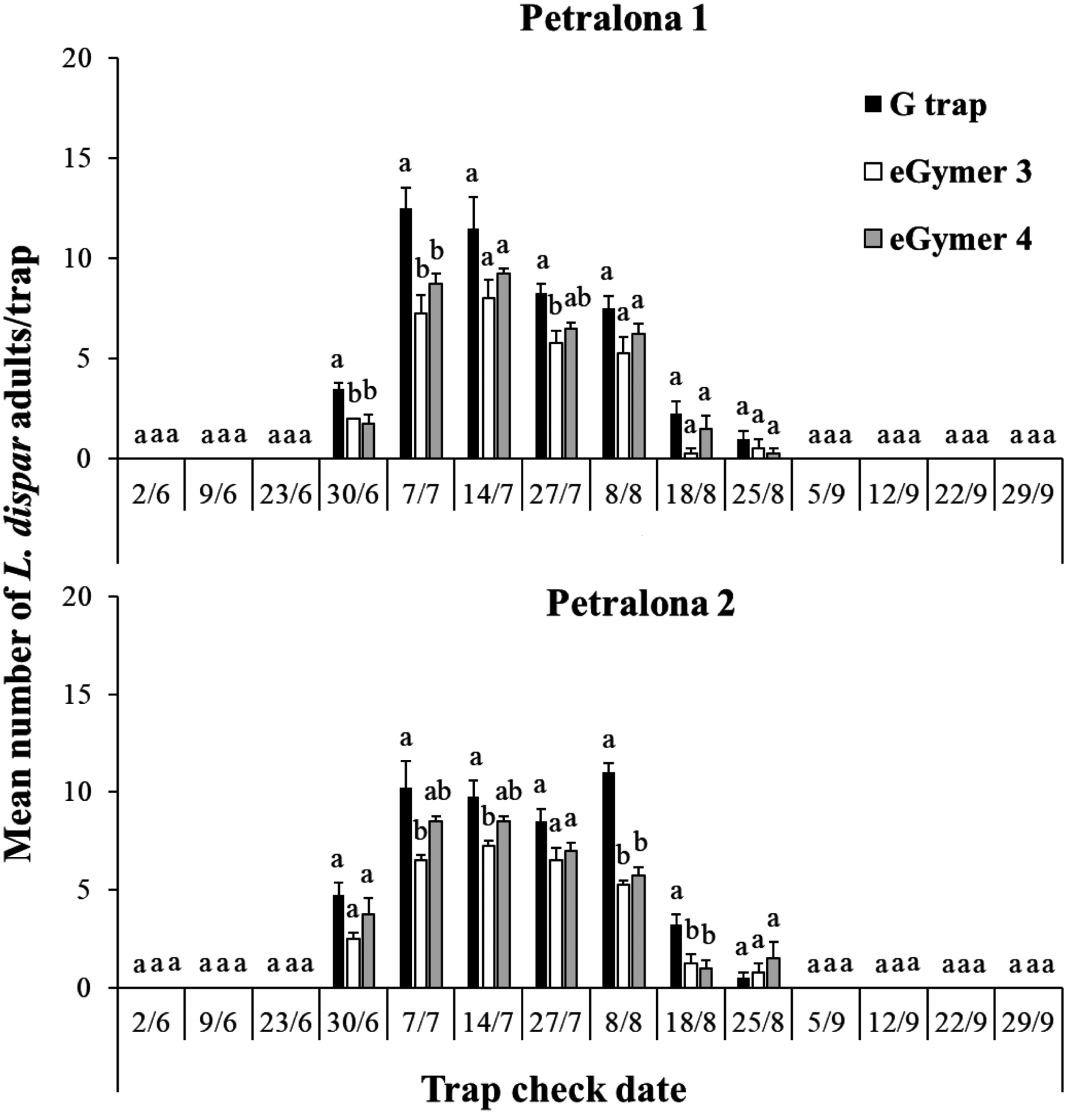
Mean number (±SE) of male adults of *Lymantria dispar* captured in each trap type and each collection date in Petralona 1 and Petralona 2 for 2023 experimental period. Within each collection date, means followed by the same letter were not significantly different (in all cases *df* = 2.11). ANOVA parameters for Petralona 1 at 30/6 were: F = 8.60, *P < *0.01, at 7/7 were: F = 9.93, *P < *0.01, at 27/7 were: F = 6.97, *P = *0.01, for Petralona 2 at 7/7 were: F = 5.57, *P = *0.02, at 14/7 were: F = 5.35, *P = *0.02, at 8/8 were: F = 66.40, *P < *0.01, at 18/8 were: F = 7.30, *P = *0.01.

In total, 439 and 456 *L. dispar* were captured in trap types at Petralona 1 and Petralona 2 in 2023 (Fig. 1). For both areas, the flight period started the last week of June and lasted until the end of August (Fig. 6). The G trap captured significantly more *L. dispar* in 3 out of 14 collection dates in Petralona 1 and in 4 out of 14 collection dates in Petralona 2. Among them, eGymer 4 captured similar abundances of *L*. *dispar* as the G trap in 1 out of 3 collection dates in Petralona 1, and in 2 out of 4 collection dates in Petralona 2 (Fig. 6).

### Complete Trap Comparison

#### Experimental Period 2022

In total, 25, 21, 73, 33, 590, 521, 593, and 593 male adults of *L. dispar* were captured in Kouri, Portaria, Lykodromio, Stavroupoli, Kryoneri cemetery, Kryoneri 1, Kryoneri 2, and Saint Nikolaos, respectively (Fig. 1). According to the trap captures, the activity of *L. dispar* seemed to be more intense in Southern Greece (Kryoneri cemetery, Kryoneri 1, Kryoneri 2, and Saint Nikolaos) (total captures ranged from 521 up to 593) than in Central and Northern Greece (Kouri, Portaria, Lykodromio, and Stavroupoli) (total captures ranged from 21 up to 73). In all areas, the four distinct trap catch rates fluctuated at similar levels, except for Kryoneri 1, where G trap and eGymer 3 captured a mean number of 15 adults per trap in contrast to eGymer 1 and eGymer 2, which captured a mean number of 4.30 and 5.69 adults per trap, respectively (Table 4). G trap in Kryoneri cemetery and Saint Nikolaos captured around 16 adults per trap, while the same trap in Kouri, Portaria, Lykodromio, and Stavroupoli gathered a low number of *L. dispar* males (0.40–1.40 adults per G trap) (Table 4). eGymer 1 captured similar numbers of *L. dispar* adults in all experimental areas, while eGymer 2 collected a mean number of 8.46 adults in Kryoneri 2 and Saint Nikolaos in relation to Kouri, Portaria, Lykodromio, and Stavroupoli where the captures ranged from 0.10 up to 1 adult per trap. In addition, eGymer 4 captures in Kryoneri cemetery, Kryoneri 1, Kryoneri 2 and Saint Nikolaos fluctuated from 14.84 up to 15.61 adults per trap in relation to Kouri, Portaria, Lykodromio, and Stavroupoli, where the captures ranged from 1 up to 2.30 adults (Table 4).

**Table 4.** Mean number (±SE) of male adults of *Lymantria dispar* captured in each trap type in experimental sites located in Greece for the 2022 and 2023 experimental periods (complete trap comparisons)

	G trap		eGymer 1	eGymer 2	eGymer 3	eGymer 4	eGymer 5	eGymer 6
Sites	2022	2023	2022	2022	2022	2023	2023	2023
Kouri	0.40 ± 0.40	0.00 ± 0.00	0.20 ± 0.20	0.70 ± 0.70	1.20 ± 1.20	0.36 ± 0.20	0.00 ± 0.00	0.09 ± 0.09
Portaria	0.10 ± 0.10	0.00 ± 0.00	0.90 ± 0.60	0.10 ± 0.10	1.00 ± 0.53	0.00 ± 0.00	0.00 ± 0.00	0.00 ± 0.00
Lykodromio	1.40 ± 0.70	10.33 ± 6.21	2.60 ± 1.35	1.00 ± 0.51	2.30 ± 1.34	2.91 ± 1.10	0.41 ± 0.28	1.00 ± 0.44
Stavroupoli	0.50 ± 0.22	6.50 ± 2.98	1.40 ± 0.76	0.40 ± 0.22	1.00 ± 0.68	2.08 ± 0.90	0.66 ± 0.28	1.16 ± 0.50
Kryoneri Cemetery	16.00 ± 3.94	–	6.00 ± 2.53	7.76 ± 2.85	15.61 ± 3.88	–	–	–
Kryoneri 1	15.00 ± 3.64	2.64 ± 0.84	4.30 ± 1.67	5.69 ± 1.93	15.07 ± 3.74	1.57 ± 0.66	0.07 ± 0.07	2.14 ± 0.79
Kryoneri 2	15.76 ± 4.14	2.71 ± 0.89	6.53 ± 2.87	8.46 ± 3.22	14.84 ± 3.97	1.64 ± 0.57	0.21 ± 0.11	2.71 ± 0.98
Saint Nickolaos	15.92 ± 4.31	–	5.76 ± 2.30	8.46 ± 3.01	15.46 ± 4.63	–	–	–

–Sites where traps were not installed for the 2023 experimental period.

#### Experimental Period 2023

**Fig. 7.**
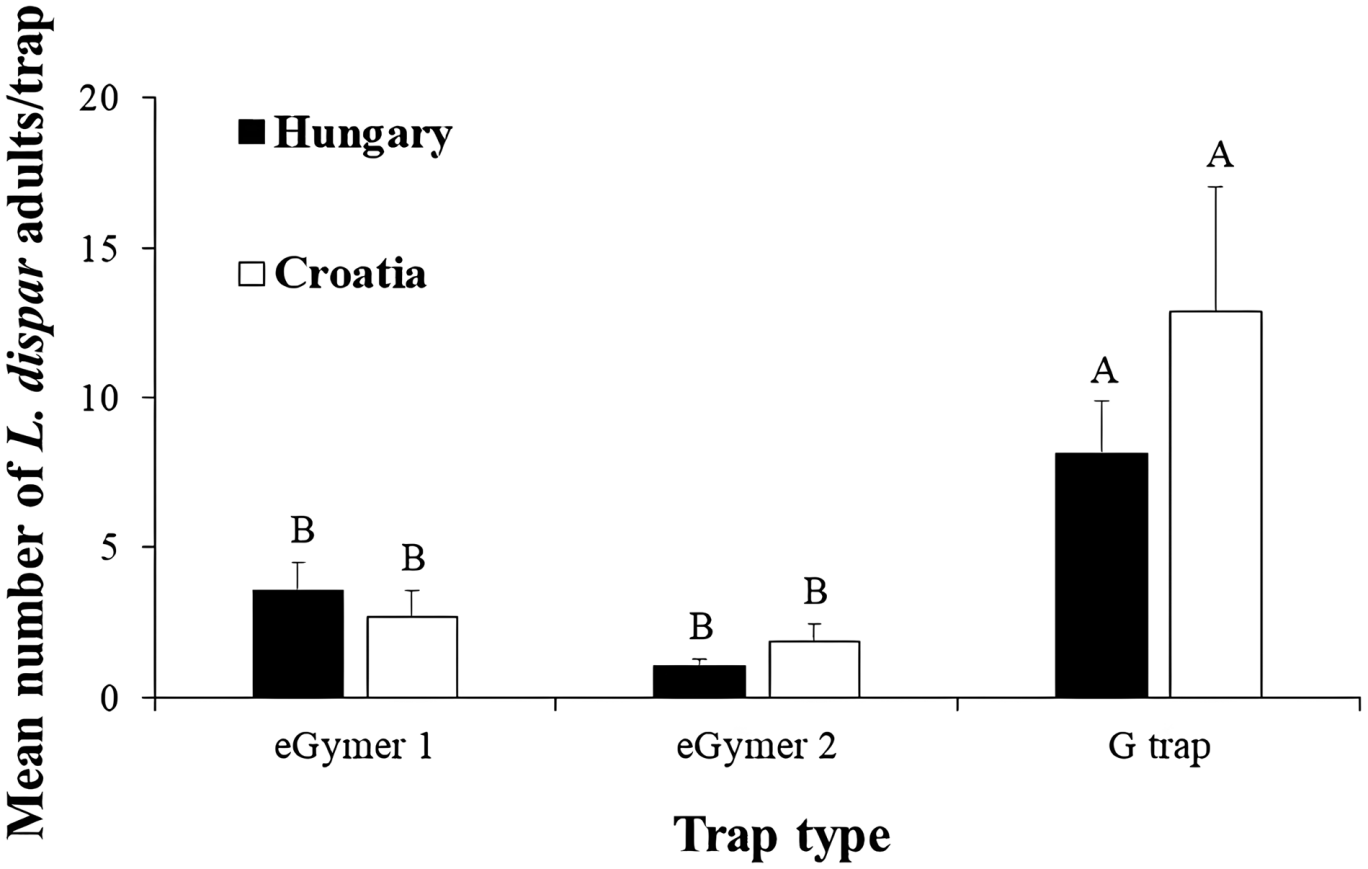
Mean number (±SE) of male adults of *Lymantria dispar* captured in each trap type in two experimental sites located, one in Hungary (Telki) and one in Croatia (Koška) for the 2023 experimental period (complete trap comparison). Within each experimental site, means followed by the same letter were not significantly different (in all cases *df = *2.116). Where no letters exist, no significant differences were noted. ANOVA parameters for Hungary were: F = 9.69, *P < *0.01, for Croatia, were: F = 5.98, *P < *0.01.

In total, 5, 0, 176, 125, 90, 102, 40, and 72 male adults of *L. dispar* were captured in Kouri, Portaria, Lykodromio, Stavroupoli, Kryoneri 1, Kryoneri 2, Telki, and Koška, respectively (Fig. 1). The population of *L. dispar* was higher in Lykodromio and Stavroupoli in relation to Kouri, Portaria, Kryoneri 1, Kryoneri 2, Telki, and Koška, while the lowest mean number of captures was observed in Portaria and Kouri with an average of 0 and 0.3, 0, 0.09, and 0 adults per trap for eGymer 4, eGymer 5, eGymer 6, and G trap, respectively (Table 4 and Fig. 7). The G trap captured more adults compared to eGymer 1 and eGymer 2 both in Telki and Koška (Fig. 7). eGymer 6 captured similar mean numbers of *L. dispar* adults in all experimental sites. The G trap had zero captures in Kouri and Portaria and 10.33 adults per trap in Lykodromio, while zero captures were also noted for eGymer 4 in Portaria. Captures of eGymer 5 did not exceed 0.66 adults per trap for any of the experimental sites (Table 4). The correlation coefficient values were positive and significant for all pairs of traps for Telki and Koška (Table 5).

**Table 5.** Correlation coefficient values (*r*) for captures between pairs of different trap types in Hungary and Croatia during the 2023 experimental period (complete trap comparison) (in all cases *df* = 38)

	Hungary		Croatia	
	Telki		Koška	
Pair of traps	R	*P*	R	*P*
G trap—eGymer 1	0.35^a^	**0.02**	0.85^a^	**<0.01**
G trap—eGymer 2	0.79^a^	**<0.01**	0.49^a^	**<0.01**
eGymer 1—eGymer 2	0.43^a^	**<0.01**	0.70^a^	**<0.01**

^a^An asterisk declares that value is significantly different from 0, two-tailed *t* test at 0.05.

## Discussion

In this study, we examined different trap designs for capturing *L*. *dispar* across a large geographic area in Europe. These data were then used to describe flight periodicity and to compare captures among collection dates at each site. To our knowledge, this is the only simultaneous, coordinated, area-wide monitoring of *L. dispar* in different European countries. Earlier studies have illustrated that *L*. *dispar* occurs in a wide variety of habitats in Europe (Doane and McManus 1981, Lance 1983, Lechowicz and Mauffette 1986, Shields et al. 2003).

The flight periodicity of *L. dispar* in Slovenia, Spain, and Greece exhibited a consistent pattern, typically starting towards the end of June and extending until mid-August. In Slovenia, the flight period began at the end of June and lasted through mid-August in both 2022 and 2023, showing little variation between years. In Spain, the flight period started slightly earlier, around mid-June in 2022, and continued until early August, with a similar pattern observed in 2023, although it began slightly later in June. Notably, *L. dispar* flight activity in Spain spanned the summer months more fully, indicating a broader range of environmental conditions that supported moth activity. In Greece, the flight activity also commenced at the end of June, continuing through mid-August, but with some regional variation in duration. For instance, in Drama, the flight ended by late July in 2022, while in Pindos, it extended until mid-August. Overall, the flight periods across these regions predominantly fell within the summer months, peaking between late June and early August, suggesting a common response to regional climatic conditions that support the lifecycle of *L. dispar* during this time.

Detection sensitivity is the cornerstone of insect trapping (Athanassiou et al. 2002, 2004, Cardé et al. 2018, Karakasis et al. 2021, Karakantza et al. 2023, Kavallieratos et al. 2023). Our data show that, in some of the areas tested, some trap types were able to capture *L. dispar* earlier than others at the beginning of the flight period. For instance, in 2023 in Ginjevec 2, eGymer 3 detected *L. dispar* seven days earlier than the G trap, even though the G trap overall captured higher abundances. This is particularly important in areas that have not been colonized yet by *L. dispar*, where detection sensitivity is desired more than the overall capacity of a given trap type to capture *L*. *dispar*. Nevertheless, in other areas, such as in Petralona 1 and Petralona 2 in 2023, all traps tested provided similar and “synchronized” results in terms of flight initiation and termination. This “synchronization” of the traps is also an important characteristic, for example when using data from different trap types to schedule control measures (Athanassiou et al. 2002, 2004, Karakasis et al. 2021, Karakantza et al. 2023).

Male adult activity, at least as this is depicted here through captures in pheromone-baited traps, lasted about two or three weeks longer in some areas than others. Similar fluctuations have been reported in the case of *T. pityocampa*, where the flight period in certain areas of Greece was much longer than in Italy and Spain (Athanassiou et al. 2017). Further analyses of these data for *T. pityocampa* revealed the occurrence of the mitochondrial lineage *T. pityocampa* “ENA clade” (Avtzis et al. 2018) and also a different species, the Cyprus processionary caterpillar, *Thaumetopoea wilkinsoni* Tams (Lepidoptera: Thaumetopoeidae) (Kerdelhué et al. 2015). Interestingly, both *T. pityocampa* and *T. wilkinsoni* use the same sex pheromone (Battisti et al. 2015). These types of relationships merit investigation in *L*. *dispar*, as different subspecies of *L*. *dispar* overlap in their distribution in Europe (Reineke and Zebitz 1998, Pogue and Schaefer 2007).

Overall, we documented that, among the traps tested, the G trap was most effective. However, some of the datasets from Spain indicated that the G trap was indeed more effective for capturing *L*. *dispar* when captures of *L. dispar* were high, while these differences declined when captures were low. Similar results were observed in Slovenia in 2022 and 2023. Among other factors, these dissimilarities could be attributed to differences in climate, microclimate, and forest conditions (e.g., stand structure and composition) among locations. The latter is particularly important in terms of its influences on trap apparency and thus trap performance. In an earlier study for *T. pityocampa*, Athanassiou et al. (2007) found that the density of pines was a determinative factor affecting trap performance. In that study, significant differences were observed between captures of traps with sticky surfaces in low-density stands while no differences were observed in medium- and high-density stands (Athanassiou et al. 2007). We hypothesize that parameters like tree density, tree size, and plant species composition played an important role in trap performance for *L*. *dispar*, along with other abiotic factors (Barbosa and Greenblatt 1979, Gottschalk et al. 1990).

Taking into account the size of *L*. *dispar*, we believe that non-sticky traps are more adaptable as sticky surfaces may become saturation with moths, moth scales and foreign materials, while perhaps influencing attraction due to adhesion of pheromones or release of volatiles from sticky materials. Earlier experiments proved that funnel (bucket) traps are superior to adhesive traps for *L*. *dispar* (Cardé et al. 2018). In contrast, funnel traps were proved to be inferior to sticky traps for capturing *T. pityocampa* (Athanassiou et al. 2007). The traps used in our experiments were based on a similar “funnel” concept, with one major difference: in some of the traps the insects had to move downwards to be captured (one hole), while in others there was only a horizontal movement (two holes). Despite the fact that there was some considerable variability in trap catches, the one-hole trap was proved to be the most effective, probably due to the reduced chance for escapees. This observation is also supported by the introduction of an insecticide as a killing agent in the 2023 experiments, which improved the performance of the horizontal movement traps. However, the occurrence of an insecticide in a trapping device increases the need for special care by the operators to avoid direct exposure to the toxicant. Our data can be used to illustrate the occurrence and distribution of *L. dispar* in a range of European countries, especially in Southern Europe, for which there is not much data available. Considering the epidemic outbursts of *L*. *dispar* and European Union directives to mitigate deforestation (Cesar de Oliveira et al. 2024), the data presented herein can be adapted to harmonizing monitoring protocols for *L*. *dispar* using standardized trap designs across large geographical areas.
